# Confirmation and expansion of the phenotype of the *TCEAL1*-related neurodevelopmental disorder

**DOI:** 10.1038/s41431-023-01530-6

**Published:** 2024-01-10

**Authors:** Fatimah Albuainain, Yuwei Shi, Sarah Lor-Zade, Ulrike Hüffmeier, Melissa Pauly, André Reis, Laurence Faivre, Julien Maraval, Ange-Line Bruel, Frédéric Tran Mau Them, Tobias B. Haack, Ute Grasshoff, Veronka Horber, Rachel Schot, Marjon van Slegtenhorst, Martina Wilke, Tahsin Stefan Barakat

**Affiliations:** 1 Department of Clinical Genetics, Erasmus MC University Medical Center, Rotterdam, The Netherlands; 2 Institute of Human Genetics, Universitätsklinikum Erlangen, Friedrich-Alexander-Universität Erlangen-Nürnberg, 91054 Erlangen, Germany; 3 Centre for Rare Diseases Erlangen (ZSEER), Erlangen, Germany; 4 Centre de Génétique et Centre de Référence Anomalies du Développement et Syndromes Malformatifs, Fédération Hospitalo-Universitaire TRANSLAD et Institut GIMI, Dijon Bourgogne University Hospital, F-21000 Dijon, France; 5 Inserm UMR1231 team GAD, University of Burgundy and Franche-Comté, F-21000 Dijon, France; 6 Centre de Référence Déficiences Intellectuelles de causes rares, Dijon Bourgogne University Hospital, F-21000 Dijon, France; 7 Functional Unit of Innovative Diagnosis for Rare Diseases, Dijon Bourgogne University Hospital, F-21000 Dijon, France; 8 Institute of Medical Genetics and Applied Genomics, University of Tübingen, Tübingen, Germany; 9 Centre for Rare Diseases, University of Tübingen, Tübingen, Germany; 10 Department of Pediatric Neurology and Developmental Medicine, University Children’s Hospital Tübingen, Tübingen, Germany; 11 Discovery Unit, Department of Clinical Genetics, Erasmus MC University Medical Center, Rotterdam, The Netherlands; 12 ENCORE Expertise Center for Neurodevelopmental Disorders, Erasmus Medical Center, Rotterdam, The Netherlands

**Keywords:** Genetics research, Genetics

## Abstract

Numerous contiguous gene deletion syndromes causing neurodevelopmental disorders have previously been defined using cytogenetics for which only in the current genomic era the disease-causing genes have become elucidated. One such example is deletion at Xq22.2, previously associated with a neurodevelopmental disorder which has more recently been found to be caused by de novo loss-of-function variants in *TCEAL1*. So far, a single study reported six unrelated individuals with this monogenetic disorder, presenting with syndromic features including developmental delay especially affecting expressive speech, intellectual disability, autistic-like behaviors, hypotonia, gait abnormalities and mild facial dysmorphism, in addition to ocular, gastrointestinal, and immunologic abnormalities. Here we report on four previously undescribed individuals, including two adults, with de novo truncating variants in *TCEAL1*, identified through trio exome or genome sequencing, further delineating the phenotype of the *TCEAL1*-related disorder. Whereas overall we identify similar features compared to the original report, we also highlight features in our adult individuals including hyperphagia, obesity, and endocrine abnormalities including hyperinsulinemia, hyperandrogenemia, and polycystic ovarian syndrome. X chromosome inactivation and RNA-seq studies further provide functional insights in the molecular mechanisms. Together this report expands the phenotypic and molecular spectrum of the *TCEAL1*-related disorder which will be useful for counseling of newly identified individuals and their families.

## Introduction

Deletions at chromosome Xq22.2 have been associated with a neurodevelopmental disease trait, presenting amongst other features with developmental delay (DD), intellectual disability (ID), behavioral abnormalities and hypotonia [1, 2]. While the disease-causing genes within the Xq22.2 deletion region remained a longstanding mystery [2–4], Hijazi et al. recently described six individuals harboring de novo variants in the *TCEAL1* gene. This single coding-exon gene located at Xq22.2 encoding a nuclear phosphoprotein referred to as TCEAL1 (or p21/SIIR) [5], likely explains some of the phenotypes caused by Xq22.2 deletions. Affected individuals presented with a variety of clinical features, including DD/ID, speech delay, autism spectrum disorder behaviors, hypotonia, gait abnormalities, seizures, brain and ocular anomalies, gastrointestinal symptoms, and recurrent infections. Mild dysmorphic craniofacial features included brachycephaly, mild facial coarsening, a broad forehead, deep-set eyes, telecanthus, a prominent bow shaped upper lip and slightly low set ears. The combination of symptoms clinically overlaps with those of individuals carrying a Xq22.2 deletion.

Given that so far only six individuals with de novo *TCEAL1* variants have been described, the full phenotypic spectrum of the *TCEAL1*-related disorder remains to be defined. Here, we report four previously undescribed individuals with de novo variants in *TCEAL1*, exhibiting intellectual disability, behavioral complaints, and dysmorphic features, clinically reminiscent of the previously described individuals. This case series includes two adults exhibiting additional phenotypes, not highlighted in the earlier pediatric cohorts, further delineating the clinical spectrum of this recently described disorder (OMIM 301094).

## Methods

### Patient recruitment and genomic investigations

All affected individuals were investigated by their referring physicians and all genetic analyses were performed in a clinical diagnostic or research setting at the local sites. Individual 1 was diagnosed with the *TCEAL1*-related disorder upon diagnostic trio exome sequencing investigations performed at the Clinical Genetics Department of Erasmus MC University Medical Center Rotterdam, performed as previously described [6]. Subsequently, the three other reported individuals were recruited through our international collaborative network and the web-based tool GeneMatcher [7]. Legal guardians of affected individuals gave written informed consent for publication of anonymized medical data and clinical photographs, obtained through the referring physicians in each collaborating center, in accordance with the declaration of Helsinki.

### Clinical reports

#### Individual 1

**Fig. 1:**
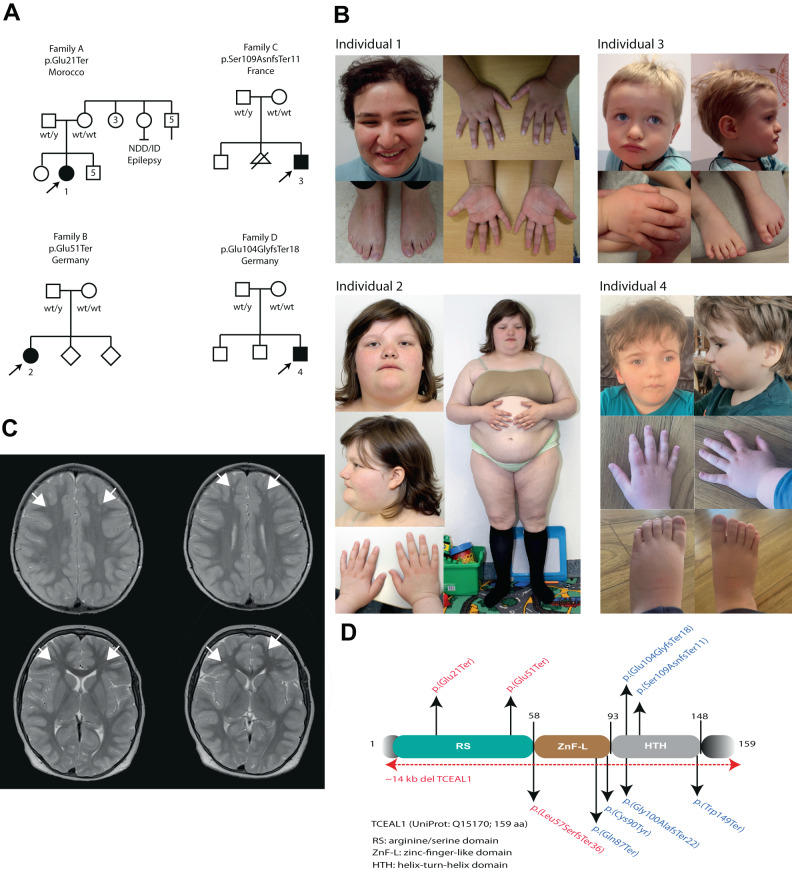
Clinical phenotypes of individuals with *TCEAL1* variants. **A** Family pedigrees of affected individuals, with *TCEAL1* variant indicated. Affected individuals harboring *TCEAL1* variants are indicated with black filled symbols, numbered, and highlighted by the arrows. **B** Clinical photographs of affected individuals. Individual 1 has a coarse face, with straight eyebrows, deep-set and elongated palpebral fissures, prominent nasal tip, short philtrum, pointy chin, and a high anterior hairline. Hands with short appearing distal phalanges and mild bilateral fifth digit clinodactyly. Feet with hallux valgus. Individual 2 has deep-set and long palpebral fissures with mild downslant, a broad nasal bridge, thin and slightly tented upper lip vermilion, small ears, hands with short appearing distal phalanges and flat feet. Individual 3 has a triangular face, with a high and wide forehead, frontal bossing, hypertelorism, downslanting palpebral fissures, hypoplasia of the alae nasae, short philtrum and thin upper and lower lip vermilion. Feet are flat and in valgus position. Individual 4 has a high anterior hairline and frontal bossing, deep-set and elongated palpebral fissures with mild ptosis, bulbous nose with a flattened nasal bridge and round nasal tip, hypertelorism, micrognathia, brachydactyly and low-set ears. **C** T2 weighted brain MRI images from Individual 2 showing bilateral frontal focal subcortical heterotopia (indicated with arrow heads). **D** Schematic representation of the TCEAL1 protein (UniProt: Q15170, 159 aa). Three domains are indicated: the arginine/serine (RS) rich domain (green), the zinc finger-like (ZnF-L) domain (brown), and the helix-turn-helix (HTH) domain (grey). Variants reported in this paper (above), and the previously reported de novo variants in TCEAL1 [5] (below) are indicated. In red are variants found in females and in blue are variants found in males. Red dotted line represents the previously reported ~14 kb *TCEAL1* deletion.

Individual 1 is a currently 22-year-old female that presented, with intellectual disability, behavioral concerns and obesity (Fig. 1A). She was born as the fourth of seven children to non-consanguineous Moroccan parents. Her family history is unremarkable, with the exception of a maternal aunt (A:I-8) that is said to have epilepsy and intellectual disability, but her medical data is unavailable.

The pregnancy and delivery were uneventful, with a birth weight of 3200 g (0 SD). There were no concerns during her first year. Independent ambulation was achieved by 14-months. Speech delay was subsequently noticed, with first words at 3 years of age, and ability to speak full sentences at age of 7 years. ENT investigations showed a moderate conductive hearing loss (30 decibels right,15 decibels left), which improved upon placement of tympanostomy tubes and adeno-tonsillectomy. From 6 years of age, she followed special education. At the age of 10 years, an overweight girl with an inappropriate happy demeanor was seen with a height of 144.5 cm (0 SD) and a weight of 53.8 kg (weight to height, + 3.2 SD; BMI, 28 kg/m^2^). Physical examination and neurological examination showed no major abnormalities other than speech delay. A brain CT-scan was unremarkable. Her behavior was described as difficult, impulsive, quick to anger, creating messes, pulling her hair, continuously and secretly eating, showing psychotic behavior including talking to the wall and hearing voices, and sleep disturbance. Contact with other children was challenging, including verbally and physically aggressive behavior, leading to suspension from school at age of 8 years. She was prescribed risperidone to control her behavioral issues, which was unsuccessful. Also, olanzapine and pipamperone had no effects on her behavioral anomalies. Esophagus contrast imaging confirmed gastro-esophageal reflux (GERD) and weight reduction was advised. Around 10 years of age, ophthalmological investigations revealed a right-sided old macular scar suspecting but not confirming congenital toxoplasmosis infection. Furthermore, idiopathic bilateral chorioretinitis and uveitis were found. Menarche occurred at 14 years of age. She was then described as physically healthy and strong, although unmotivated to perform tasks. Her weight gain had reached obesity levels. No seizures occurred. Neurological examination at 19 years of age, including EEG, showed no abnormalities. An MRI showed no signs of gross structural brain alterations.

**Fig. 2:**
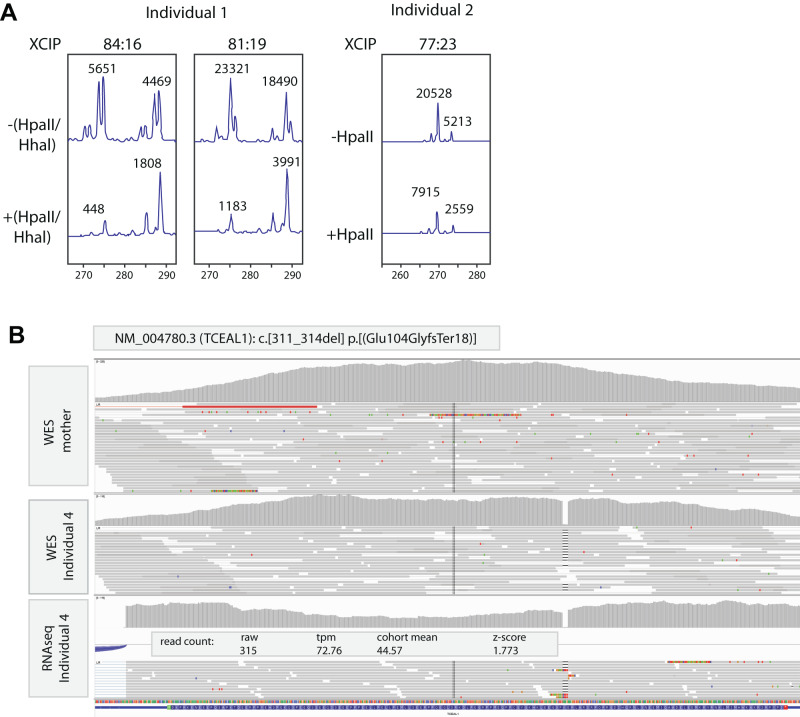
X chromosome inactivation and RNA-seq studies. **A** Analysis of X-inactivation for individual 1 (performed twice in two different blood derived DNA samples) and individual 2, performed as previously described [10, 12]. In the undigested samples, PCR analysis visualizes the two alleles of a polymorphic short tandem repeat at the human androgen receptor locus (HUMARA). Upon digestion with the methylation sensitive restriction enzymes Hpall and Hhal, only the methylated allele on the inactive X chromosome is amplified, and the ratio between results from digested and undigested samples is used to calculate the ratio of X inactivation skewing. XCIP = X chromosome inactivation pattern; −(Hpall/Hhal) = undigested DNA; +(HpaII/Hhal) = digested DNA. **B** IGV genome browser view of exome sequencing (on blood derived DNA) from unaffected mother (upper track) and her son (individual 4, middle track) and RNA sequencing of fibroblasts derived from individual 4 (lower track). Results confirm the de novo origin of the NM_004780.3 (*TCEAL1*):c.311_314del, p.(Glu104GlyfsTer18) variant and its stable expression at RNA level with a z-score of 1.773 in fibroblasts without evidence of nonsense-mediated decay.

Investigation at the age of 22 years showed a length of 159.5 cm (−1.8 SD), obesity with 96.5 kg (weight to height, +4.65 SD; BMI, 38 kg/m^2^) and a head circumference of 55.5 cm (0 SD). Dysmorphic features included a full and coarse facial gestalt, straight eyebrows, deep set eyes with elongated palpebral fissures, a prominent nasal tip, short philtrum, pointy chin, high anterior hairline, and scarce head hair likely due to trichotillomania. Hands appeared short and broad with mild bilateral clinodactyly, and feet with hallux valgus (Fig. 1B). Metabolic screening in plasma and urine including metabolites for cerebrotendinous xanthomatosis was unremarkable. SNP-array was normal. Trio whole exome sequencing (WES), first focusing on a panel of ~1200 genes involved in intellectual disability, followed by analysis of all protein coding genes identified a de novo *TCEAL1* (NM_004780.3):c.61 G > T, p.(Glu21Ter) variant (Table S1), with no other (likely) pathogenic variant being identified. Methylation analysis of the human androgen receptor locus performed in blood derived DNA showed evidence of skewed X inactivation (84 and 81%, respectively, in two independent measurements) (Fig. 2A).

#### Individual 2

Individual 2 (Fig. 1A) is a currently 22-year-old female. She is the oldest of three children born to non-consanguineous Caucasian-German parents, who came to medical attention in infancy, presenting with developmental delay, severe intellectual disability, behavioral concerns, hypotonia and ocular anomalies. Pregnancy was complicated by preterm contractions, with a further uneventful delivery at 40 weeks of gestation (Apgar scores 10/10), with birth weight of 3210 g (−0.5 SD), length of 50 cm (−0.7 SD) and a head circumference of 34 cm (−0.7 SD). For the first two months, feeding problems occurred. Hypotonia was evident in infancy, with independent sitting and walking (with support) achieved at the age of 10 months, and 2 years, respectively. She started speaking around 3 years of age and mainly communicates with single words. In childhood, ataxic movements and stereotypes were reported. No regression occurred. Behavioral concerns included a missing feeling of satiety, a need for routines, sleeping problems and depressive episodes. Testing for autism showed unclear results. Although not formally diagnosed with epilepsy, she had two generalized seizures in infancy. At the age of 5 and 9 years, brain MRIs showed bilateral frontal focal subcortical heterotopia (Fig. 1C). Ophthalmological investigation showed strabismus, hypermetropia and astigmatism. She was diagnosed with Hashimoto thyroiditis, also present in her otherwise healthy sister. Her menarche occurred at the age of 16 years, followed by irregular menses, and she has been diagnosed with hyperandrogenemia, hyperinsulinemia and polycystic ovary syndrome (PCOS). Other findings included suspected gluten intolerance (not confirmed by laboratory testing), reduced pain sensation and intermittent urinary incontinence.

At the last investigation at age of 22 years, her length was 150.2 cm (−3.3 SD) with a weight of 90 kg (weight to height, +4 SD; BMI of 39.9 kg/m^2^) and a head circumference of 54.5 cm (0 SD). She shows psychomotor delay in her fine and gross motor skills, and intellectual disability. Her latest IQ test scored < 30, which might have been an underestimation given her behavior complicating the testing. She cannot read but recognizes words and can follow an easy conversation when provided with structures. She is difficult to understand, and her passive language is reported to be better than her expressive language. Currently her gait is slow, she needs orthotic insoles for flat feet, and has poor coordination when climbing stairs. Dysmorphic features include deep-set and elongated palpebral fissures with mild down-slant, a broad nasal bridge, thin and slightly tended upper lip vermilion, small ears, hands with short appearing distal phalanges and flat feet (Fig. 1B).

Genetic investigations included a normal karyotype, normal Fragile X syndrome testing, and a normal SNP-array. Other targeted investigations included tests for Angelman, Prader Willi, Cohen and Smith Magenis syndrome, which were unremarkable. WES revealed a heterozygous de novo single nucleotide stop variant in *TCEAL1* (NM_004780.3):c.151 G > T, p.(Glu51Ter), with no other (likely) pathogenic variants detected (Table S1). X-inactivation analysis showed a near borderline skewing, with an average of 77% preference to one of the X-chromosomes being inactivated (Fig. 2A).

#### Individual 3

Individual 3 (Fig. 1A) is a currently 4 years and 7 months old boy with severe global developmental delay of motor and language skills, hypotonia and global hyperlaxity, who first came to medical attention at the age of 8 months. He is the second child of non-consanguineous Caucasian parents with an older healthy brother. One miscarriage of parents occurred between the first and second child. The pregnancy and delivery were uneventful (Apgar scores 7/10), with a birth length of 47 cm (−0.75 SD), weight of 2840 g (−1.1 SD) and head circumference of 34.5 cm (−0.5 SD). A brain MRI was conducted at the age of 14 months, showing no abnormalities.

At the time of the last investigation, his length was 102.5 cm (−2 SD), with weight of 16.5 kg (0 SD), and head circumference of 52.2 cm (0 SD). No official IQ testing has been performed, but based on clinical grounds his functioning is estimated to reflect severe to profound ID. He has no verbal skills and only produces sounds, but can recognize his name, play simple games and shows facial expressions for “like” and “dislike”. He makes normal eye contact and shows adaptive behavior. No neurodevelopmental regression, psychiatric disorders, or epilepsy occurred. Generalized joint laxity without joint luxations was noted. Given his severe hypotonia, he requires a corset and equipment to stay seated if not in a tripod position and is non-ambulatory. He can roll over from his back and raise his head and shoulders once he is in ventral position and is able to hold his head up. Hearing is normal. Ocular testing found anisocoria, hypermetropia, intermittent bilateral strabismus and astigmatism. Cardiac ultrasound and chest X-ray were normal. Constipation was successfully treated with laxatives. Continence was not yet achieved. Dysmorphic facial features comprise a triangular face shape, high and wide forehead, frontal bossing, hypertelorism, down-slanting palpebral fissures, hypoplasia of the alae nasae, short philtrum, and thin upper and lower lip vermilion (Fig. 1B). Other findings included a postural kyphosis and flat feet in valgus rotation.

Genetic investigations included a normal karyotype, normal Fragile X syndrome testing, and a normal SNP-array. Routine metabolic investigations, including chromatography for amino acids in plasma and urine, were unremarkable. Initial trio WES in 2019 and trio whole genome sequencing in 2021 did not identify any likely known disease cause. Subsequently, a re-analysis focusing on recently identified new disease genes in currently unexplained individuals revealed a heterozygous de novo *TCEAL1* (NM_004780.3):c.324_333del, p.(Ser109AsnfsTer11) variant, predicting a frameshift and premature stop codon (Table S1). The variant was in retrospect first identified in the exome sequencing datasets and confirmed in the genome sequencing data, with currently no other (likely) pathogenic variants relevant for the phenotype being identified.

#### Individual 4

Individual 4 (Fig. 1A) is a currently a 6-year-old boy who was born at term as the third child of non-consanguineous Caucasian parents, with a weight of 3210 g (−1 SD), length of 52 cm (−0.4 SD), and head circumference of 34 cm (−0.7 SD), that presented with developmental delay, only starting assisted walking at the age of 3 years. Reduced fetal movements during an otherwise uneventful pregnancy were observed. After an early postnatal period with failure to thrive, the individual developed obesity at 3 years of age. The parents reported increased sweating and an overall reduced activity level as well as periods of possible developmental regression following febrile illnesses. Further development was characterized by severe intellectual disability with no active language at 5.5 years of age, self-aggressive behavior and autistic features. With his hypotonia improving after the age of two years, his mobility still seemed clumsy, possibly due to cognitive impairment and lack of attention. Brain MRIs at age 1 and 3 years showed non-specific anomalies with delayed myelination. Measurements at the last examination at 5 years and 7 months showed a length of 110 cm (−1.4 SD), weight of 25.2 kg (weight to height, +3 SD; BMI of 20.82 kg/m^2^) and a head circumference of 53.5 cm ( + 2 SD). Physical examination revealed very fair hair and translucent skin, as well as muscular hypotonia and poor eye contact. Mild dysmorphic features include a high anterior hairline and frontal bossing, deep-set and elongated palpebral fissures with mild ptosis, bulbous nose with a flattened nasal bridge and round nasal tip, hypertelorism, low-set ears and brachydactyly (Fig. 1B).

After an unremarkable SNP array analysis and Fragile X syndrome testing, trio WES was performed as previously described [8]. Initially, clinical variant prioritization failed to identify likely clinically relevant causes in genes with an established gene-disease association. However, a hemizygous de novo change in *TCEAL1* (NM_004780.3):c.311_314del, p.(Glu104GlyfsTer18), predicted to result in a frameshift and prematurely truncated protein was identified upon re-analysis (Table S1). A transcriptome analysis performed as previously described [9], on RNA derived from fibroblasts indicated that the mutant transcript was stably expressed (z-score 1.773) and apparently not subject to nonsense-mediated decay (Fig. 2B), as expected for a truncating variant in a single coding exon gene.

## Discussion

Here we report four individuals with protein truncating variants in *TCEAL1*, located at Xq22.1, presenting with developmental delay, intellectual disability, autistic-like behaviors, hypotonia, gait abnormalities and craniofacial dysmorphisms. Previously, several studies reported Xq22.2 deletions encompassing *TCEAL1* and other nearby genes [1, 2, 4], but only one study reported six individuals harboring specific de novo *TCEAL1* variants, including four protein truncating variants, one missense variant and a ~ 14 kb single gene deletion affecting *TCEAL1* [5]. The herein identified *TCEAL1* variants are novel, predicted to be pathogenic and absent in gnomAD (Fig. 1D). Affected individuals share reminiscent clinical features, including global developmental delay (4/4), severe intellectual disability (4/4), especially regarding expressive language, and behavior problems including autistic-like behavior (e.g. need for structure/routine) or verbal aggression (Table 1). Hypotonia (5/5) and gait disturbance or non-ambulation (6/6) were previously frequently reported. Similarly, three individuals from our cohort showed hypotonia, while only individual 1 had normal muscle tone without gait disturbance. Seizures or possible seizures are less common in our cohort (1/4) than in reported cases (3/6). Brain anomalies observed in previous cases (3/5) include abnormal myelination or thinning of the corpus callosum. In individual 2, we observed aberrant neuronal migration causing bilateral frontal focal subcortical heterotopia (Fig. 1C) and individual 4 showed delayed myelination. Previously described dysmorphic features (6/6) included a broad forehead (4/6), bow-shaped upper lips (4/6) and deep-set eyes (2/6), which are observed in our series, in 3/4, 1/4 and 2/4, respectively. Other frequently occurring features both in our cohort and previously described individuals included ocular anomalies, including strabismus, astigmatism, myopia or hypermetropia (previously, 6/6; here, 4/4) and gastrointestinal symptoms including GERD and constipation (previously, 3/6; here, 4/4). Recurrent infections were frequently described in the previous series (5/6) but not in our cohort. Interestingly, obesity was observed in three individuals here, and in two previously described individuals (Table 1). In the currently known individuals, obesity seemed more frequent in females (3/4) compared to males (2/6). However, we cannot exclude that differences in age can explain this sex-linked difference, as the two obese females (individual 1 and 2) described here were older at time of latest investigation. The third previously reported obese female was 6- years of age presenting with hyperphagia, obesity and premature puberty with gynecomastia [5]. Besides, individual 2 showed distinct endocrine levels with hyperinsulinemia, hyperandrogenemia, oligomenorrhea and PCOS. It is possible that these findings are secondary to her obesity. Her IGF1 levels were in the normal lower boundary range in childhood, with similar low IGF1 levels also observed in individual 3 described by Hijazi et al. [5]. Although it is tempting to speculate that these features may occur more frequently in affected females than in males, given the small cohort size this will require reporting of additional cases in the future to further clarify this.

**Table 1:** Clinical features and their frequency in individuals with *TCEAL1* variants, from our cohort and the previous study from Hijazi et al. [5], also stratified per sex.

Clinical phenotype	HPO number	Affected individuals (This paper, *n* = 4)	Affected individuals (Hijazi et al. [5], *n* = 6)	Affected males (Total, *n* = 6)	Affected females (Total, *n* = 4)	Affected individuals (Total, *n* = 10)
Neurological & psychiatric features						
DD/ID	012758/ 0001249	4/4	6/6	6/6	4/4	10/10
Delayed speech development	0000750	4/4	6/6	6/6	4/4	10/10
Hypotonia	0001319	3/4	5/5	6/6	2/4	8/10
Gait disturbance	0001288	3/4	6/6	6/6	3/4	9/10
Neurodevelopmental regression	0002376	1/4	2/6	2/6	1/4	3/10
Behavioral abnormalities	0000708	3/4	6/6	5/6	4/4	9/10
Autism/autistic-like behavior	0000717	3/4	4/6	4/6	3/4	7/10
Seizures	0001250	1/4	3/6	2/6	2/4	4/10
Abnormal myelination	0012447	1/4	3/5	2/5	2/4	4/9
Subcortical heterotopia	0032391	1/4	0/5	0/5	1/4	1/9
Abnormal corpus callosum	0001273	0/4	1/5	1/5	0/4	1/9
Ocular anomalies						
Astigmatism	0000483	2/4	5/6	4/6	3/4	7/10
Nystagmus	0000639	0/4	1/6	1/6	0/4	1/10
Strabismus	0000486	2/4	5/6	4/6	1/4	5/10
Myopia/hyperopia	0000545/ 0000540	3/4	2/6	3/6	2/4	5/10
Iris coloboma	0000612	0/4	1/6	1/6	0/4	1/10
Dysmorphic features						
Broad forehead	0000337	3/4	4/6	6/6	1/4	7/10
Deep-set eyes	0000490	2/4	2/6	2/6	2/4	4/10
Low-set ears	0000369	1/4	2/6	2/6	1/4	3/10
Thin or bow-shaped upper lip	0000233/0002263	2/4	4/6	5/6	1/4	6/10
Abnormality of fingers or toes	0011297	3/4	2/6	2/6	3/4	5/10
Other						
Gastrointestinal abnormality	0011024	4/4	3/6	5/6	2/4	7/10
Immune system abnormality	0002715	0/4	5/6	4/6	1/4	5/10
Obesity	0001513	3/4	2/6	2/6	3/4	5/10

*DD* Developmental delay, *HPO* Human Phenotype Ontology (jax.org), *ID* Intellectual disability. Obesity, defined as a BMI at or above + 2 standard deviations from the median for the same sex and age group, by the World Health organization (WHO) [13]. Brain imaging data was only available for 9 individuals in total.

Both in our study as in the previously published cohort, males (*n* = 6) seem to be even more severely affected compared to females (*n* = 4), including the level of intellectual disability, expressive language skills and motor skills impairment. The fact that *TCEAL1* is located on the X chromosome, suggests that the remaining dosage of functional TCEAL1 might influence the disease severity, leading to milder phenotypes in females. This might be further modulated due to the effects of X chromosome inactivation (XCI) in females [10]. In agreement, we observe skewed XCI in individual 1, and similar observations have been made for a female individual described in the previous cohort [5]. Although the identity of the preferentially active *TCEAL1* allele cannot be determined using the methylation assay at the human androgen receptor, it seems likely that the mutant allele is preferentially inactivated in disease relevant tissues in mildly affected females, thereby leading to milder disease phenotypes in females with the *TCEAL1*-related disorder. Alternatively, genotype-phenotype correlations might explain the observed sex-specific disease severity, as so far, the variants identified in males are preferentially located in or near the helix-turn-helix (HTH) domain of TCEAL1, whereas variants identified in females either delete the complete gene, or are located in the N-terminal arginine/serine (RS) domain of TCEAL1 (Fig. 1D). As the majority of variants identified so far result in TCEAL1 truncations, and truncated mRNAs of *TCEAL1*, being a single coding-exon gene with all variants located in the last exon, might not be subject to nonsense-mediated decay [11], as also observed in individual 4 (Fig. 2B), it is also possible that expression of various truncated proteins might cause differences in disease mechanisms depending on which protein domains are still retained.

In conclusion, the emerging *TCEAL1*-related key clinical features include intellectual disability, developmental delay, behavioral problems, hypotonia and craniofacial dysmorphisms. Males are more severely affected compared to females in terms of the level of cognition, expressive language and motor impairment. Moreover, obesity seems to be a common symptom among both males and females. Possibly, endocrine symptoms can also be part of the phenotype. Further reporting of individuals with *TCEAL1* variants will allow delineation of genotype-phenotype correlations of this novel disorder, and ultimately functional studies of *TCEAL1* will be required to better understand the role of this gene in neuronal development.

### Supplementary information

Table S1

## Data Availability

All available clinical data are presented herein. All data generated or analysed during this study are included in this published article, with the exception of raw patient RNA-seq data and genomic sequencing data that due to privacy regulations and given consent under which affected individuals were recruited, cannot be publically made available.
