# Supplementary material for: Confirmation and expansion of the phenotype of the TCEAL1-related neurodevelopmental disorder
Source: Eur J Hum Genet. 2024 Jan 10;32(3):350–6. doi: 10.1038/s41431-023-01530-6 (PMC10923854; doi:10.1038/s41431-023-01530-6)
Supplement: Supplementary file 1 — Table S1 [file 41431_2023_1530_MOESM1_ESM.docx]

Table S1 Detailed molecular information

|  | Individual 1 | Individual 2 | Individual 3 | Individual 4 |
| --- | --- | --- | --- | --- |
| Sex | Female | Female | Male | Male |
| Ancestry | Moroccan | German | France | German |
| Consanguinity | No | No | No | No |
| Variants gDNA GRCh37(chrX): | g.102,884,905G>T | g.102,884,995G>T | g.102,885,166_102,885,175del | g.102,885,151-102,885,154del |
| Variant cDNA (NM_004780.3) | c.61G>T | c.151G>T | c.324_333del | c.311_314del |
| Variant protein | p.(Glu21Ter) | p.(Glu51Ter) | p.(Ser109AsnfsTer11) | p.(Glu104GlyfsTer18) |
| Variant type | SNV Nonsense | SNV Nonsense | Deletion Frameshift | Deletion Frameshift |
| Zygosity | Heterozygous | Heterozygous | Hemizygous | Hemizygous |
| Inheritance | De novo | De novo | De novo | De novo |
| Population frequency | NP (gnomAD) | NP (gnomAD) | NP (gnomAD) | NP (gnomAD) |
| CADD scores | 33 | 35 | 33 | 33 |
| Genetic testing performed | Trio WES | Trio WES | Trio WES and WGS | Trio WES |
| Other genetic/environmental factors contributing to phenotype | None | None | None | None |

CADD: Combined Annotation Dependent Depletion (v1.6, URL:<https://cadd.gs.washington.edu/>), WES: whole exome sequencing, WGS: whole genome sequencing, NA: not applicable, NP: not present, SNV: single nucleotide variant, WGS: whole genome sequencing, gnomAD (v2.1.1 , URL:<https://gnomad.broadinstitute.org/>).
